# Sex Differences in Childlessness in Norway: Identification of Underlying Demographic Drivers

**DOI:** 10.1007/s10680-021-09590-4

**Published:** 2021-07-23

**Authors:** Øystein Kravdal

**Affiliations:** 1 Centre for Fertility and Health, Norwegian Institute of Public Health, Oslo, Norway; 2 Department of Economics, University of Oslo, Oslo, Norway

**Keywords:** Childlessness, Women, Men, Sex difference, Demographic decomposition, Register data

## Abstract

In Norway, as in many other rich countries, childlessness is more common among men than women and has also increased more among men. Over the last 15 years, the gap in childlessness between 45-year-old women and men has widened from 5.8 to 10.2 percentage points, according to national register data. In the Norwegian-born subgroup, the gap has increased by 2.4 percentage points, from 5.8 to 8.2. The goal of the study was to identify the demographic drivers of this development, using a quite simple, but original, decomposition approach. The components reflect changes in relative cohort sizes, whether the child has one native and one immigrant parent, whether the father was older than 45, and whether one of the parents already had a child, no longer lived in Norway at age 45, or was unidentified. It was found that the modestly increasing sex gap in childlessness among the Norwegian-born is largely linked to changes in cohort sizes, i.e. fertility trends. Changes in re-partnership have actually contributed weakly in the opposite direction: It has become more common especially among men to have the first child with a partner who already had a child, and thus not contribute to bringing also that person out of childlessness. The importance of the various components is different for immigrants, among whom the sex gap in childlessness has increased particularly much. This development may also reflect that especially male immigrants perhaps have children in the home country who are not included in the Norwegian register.

**Supplementary Information:**

The online version contains supplementary material available at 10.1007/s10680-021-09590-4.

## Introduction

**Fig. 1:**
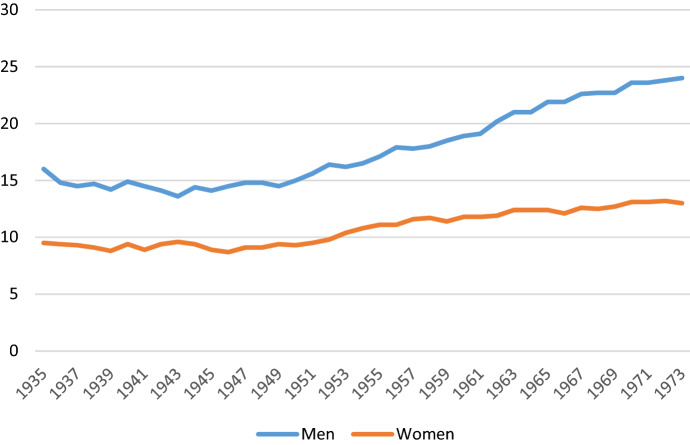
Percent childless at age 45, by sex and birth cohort, in Norway. *Note*: The numbers are calculated by the author from register data (see text for description of data) and are almost identical to those calculated from the same source and published routinely by Statistics Norway (2021)

The proportion of women who are childless at age 45 has increased over the last decades in most rich countries and is now typically between 10 and 20% (Frejka, 2017; Sobotka, 2017).^1^ This development, which has likely been driven by a number of societal changes,^2^ has given rise to concerns, as childlessness may have implications both for the individuals involved^3^ and at the aggregate^4^ level, in addition to being partly a result of conditions that may be seen as problematic themselves, such as economic uncertainty (Schmitt, 2021). The proportion childless tends to be even higher among men than women and has in some countries also increased more among men (Jalovaara et al., 2019). As an example of such a pattern, trends in childlessness among Norwegians of age 45 (after which almost no women and very few men have their first child^5^) are shown in Fig. 1.

An increasing sex difference in childlessness may have implications for society, primarily through individual-level consequences which in turn may have broader social impact. For example, childless women and men have much higher mortality and poorer health than parents, although this may be partly due to selection (Kravdal et al., 2020).^6^ Thus, one may argue that an increasing sex gap in childlessness may contribute to raise the proportion of men among those with poor health and in need of care, which it may be valuable to take into account when planning health services. Furthermore, one may speculate whether a more strongly rising childlessness among men than women might lead to a development in men’s experiences and life perspectives that differs from that among women, which may have consequences, for example, for future debates about gender equality and gender roles and ultimately the political decisions that are taken.

The goal of this study is to take a first step towards a better understanding of the forces behind the widening sex gap in childlessness by giving a quite detailed description of underlying “demographic” factors. An original decomposition approach, applied to Norwegian register data, is used as the tool for the description. Factors that turn out to be particularly important can be more thoroughly analysed in later investigations, with a focus on, for example, potential socioeconomic or ideational determinants.

The sex gap in childlessness, and its increase, may be seen as quite puzzling. Register-based calculations of a person’s number of children are, of course, based on children included in the register, and for the vast majority of these children, two parents that are also included in the register are identified. A first thought may be that, when a child has both a registered father and a registered mother, men’s and women’s registered fertility, including the probability of being childless, should be the same. Indeed, and as explained below, there are theoretical situations where such similarity exists, but in real life there are always differences. Schoumaker (2019) has explained how age differences between parents and differences in the sizes of the corresponding cohorts cause differences between women’s and men’s average number of children, but there is more to the story than this and, in particular, other factors must be brought in when the focus is on childlessness (or the parity distribution more generally). In the present study, the sex gap in childlessness is decomposed into a few demographic components that are obviously relevant. One of these reflects to large extent relative cohort sizes, while another reflects whether it is more common for women than for men to have their first child with a partner who is already a parent. The other components reflect emigration after childbearing, childbearing after age 45 for men, and the fact that one of the parents (typically the father) may not be reported. Below, these components are first explained intuitively. Subsequently, the approach is specified in more detailed, with further elaboration in the Supplementary Material.

In the future research, there may be in interest in doing a similar type of decomposition for various subgroups of the population, such as those with low, medium, or high education or those who are born abroad versus the natives. The sex gap in childlessness may vary between these subgroups, and the relative importance of the mentioned demographic components producing the sex gap may vary as well. However, an additional complication arises in such analysis because a person in one of these groups may have a child with a person in another group. In the last part of this study, distinction is made between immigrants and natives. This serves as a simple example of subgroup analysis because there are only two subgroups, rather than three or more. It is also an *important* example because earlier register-based research has revealed very high childlessness among male immigrants (Grasdal & Lommerud, 2019). This may partly be an artefact, in the sense that these men may have children they have not brought with them to Norway and who have therefore not been included in the population register. Given this possibility that immigrants contribute to an “unreasonably” large sex gap in childlessness in the national population, it would make sense to examine the situation in both subgroups, but with special attention to the natives.

## Methods and Data

### The Ideas Behind the Decomposition

It is quite common when comparing men’s and women’s cohort fertility to use a cut-off at the same age, although men can have children up to a much higher age than women. Furthermore, it makes good sense to compare women’s cohort fertility with that among men born two years earlier, because this is a common age difference between parents in rich countries (Dribe & Nystedt, 2017; Ní Bhrolcháin, 2005). Thus, one may, for example, consider the fertility among women of age 45 in cohort K (average or distribution of the number of children born up to that age) and the fertility among men of age 45 in cohort K-2. How can these numbers be equal, and how can they be different? To see that, let us first make the very hypothetical assumption that women and men in these cohorts two years apart have children only with each other, and before the man is 45 years old. In this situation, and if the cohorts are equally large at the outset and there is no mortality, emigration or immigration, the average number of children among women of age 45 in cohort K is bound to be the same as the average number of children among men of age 45 in cohort K-2.

Should it turn out that the pattern is different from this in real life, it would mean that at least one of the assumptions is not met. One factor that contributes to a difference between women’s and men’s fertility is the male surplus at birth: There are typically about 5% more boys than girls among the newborn (Guilmoto, 2009). Additionally, the number of births has gone down over time in many rich countries, so the two year older male cohorts may well be considerably more than 5% larger than the female cohorts, and men’s fertility correspondingly smaller. This is essentially the point made more thoroughly by Schoumaker (2019). Note, however, that even if the average number of children is the same among women and men (because women have all their children with two year older men and these male and female cohorts have the same size), the proportion childless may differ. For example, in the extreme hypothetical situation where all women have children, but only a few men are “recycled” as fathers, there will be no childless women and many childless men. (Multi-partner fertility among men has, in fact, become more common in several countries, including Norway (Lappegård et al., 2011), but if such re-partnering among men is accompanied by a corresponding tendency among women to have children with more than one man, a sex gap in childlessness will not necessarily arise.)

Another reason why fertility, including the special case of childlessness, may differ between women and men in the two mentioned cohorts when measurement is done at age 45, is that women may have children with men who are older than 45. These children only contribute to women’s fertility as measured at that age, not that of men. Additionally, some children included in the population register are not registered with a father, but with a mother (see further details about the data below). This may be because the father, although he lives or has lived in Norway and therefore is included in the register, has not been reported by the mother (in a few situations she may not even know who the father is). If so, the child contributes to women’s, but not men’s fertility. The fertility of men included in the register would then, in principle, be underreported, although one may argue that it is not underreported in some sort of “social” sense, since the men would typically not have any contact with these children either. This contribution to the sex gap in fertility is, in principle, set off against an opposite contribution from cases where only the father is reported, but this is much less common. A father may also be unidentified because he has never lived in the country, and therefore not been included in the register. For example, the mother may have had a child in Norway with a short-time visitor, or she may have had a child abroad and moved to Norway alone with the child, because of a divorce or for other reasons. The child contributes to the fertility of women, but not to that of men, also in such situations. Again, one could make a corresponding argument about unidentified mothers, who are far fewer. Obviously, a child without any of the parents registered will contribute to neither men’s nor women’s fertility.

Finally, when it is focussed on the fertility of individuals who are still resident in the country at age 45 (which it is reasonable to do as births after emigration would not be included in the register), there is also another contribution to the sex difference in childlessness: For example, in the hypothetical situation where women have most of their first births with men who soon afterwards leave the country, rather than with men who do not emigrate, there will be more childless men than women among those resident at age 45.

To generalize, the key issue is whether a person who becomes a parent then also, so to speak, brings a person of the other sex in the relevant population out of childlessness (rather than one who, for example, already is a parent or leaves the population after birth) and whether there is a sex difference in this tendency.

### More Detailed Specification

Two three-year cohorts of women are included in the decomposition analysis (rather than one-year cohorts, to increase the sample size): women born 1971–1973, who are the youngest observed up to age 45, and those born 15 years earlier, when there was a considerably smaller sex difference in childlessness. The childlessness in these female cohorts at age 45—among those who still lived in Norway at that age—is compared to that among 45-year-old men who were born two years earlier and who also lived in Norway at age 45.

To reflect the ideas above, the proportion of women in the 1956–1958 or 1971–1973 cohorts who have become mothers (Sw) is defined as:

$$ {\text{Sw}}\, = \,{\text{Bw}}\, + \,{\text{Cw}}\, + \,{\text{Dw1}}\, + \,{\text{Dw2}}\, + \,{\text{Dw3}}\, + \,{\text{Dw4}}. $$

Bw, Cw, and Dw1-Dw4 refer to first births with a man with the following characteristics (“lived in Norway at age 45” means “lived in Norway at the end of the year when he turned 45 or, if born after 1973, the end of 2018”):

Bw: He was 45 or younger at birth, lived in Norway at age 45, and had no older children.

Cw: Same, except that he had older children.

Dw1: He was 45 or younger at birth, did not live in Norway at age 45, and had no older children.

Dw2: Same, except that he had older children.

Dw3: He was older than 45 at birth.

Dw4: He was not identified.

The corresponding equation for the proportion of men in the two year older cohorts who have become fathers is:

$$ {\text{Sm}}\, = \,{\text{Bm}}\, + \,{\text{Cm}}\, + \,{\text{Dm1}}\, + \,{\text{Dm2}}\, + \,{\text{Dm4}}. $$

There is no equivalent of Dw3, because women very rarely give birth after age 45.

Thus, the difference between the proportion of women who are mothers and the proportion of men who are fathers can be decomposed like this for each of the two cohort groups (the oldest cohort group including 1954–1956 for men and 1956–1958 for women and the youngest including 1969–1971 for men and 1971–1973 for women):

$$ {\text{Sw}} - {\text{Sm}}\, = \,\left( {{\text{Bw}} - {\text{Bm}}} \right)\, + \,\left( {{\text{Cw}} - {\text{Cm}}} \right)\, + \,\left( {{\text{Dw1}} - {\text{Dm1}}} \right)\, + \,\left( {{\text{Dw2}} - {\text{Dm2}}} \right)\, + \,{\text{Dw3}}\, + \,\left( {{\text{Dw4}} - {\text{Dm4}}} \right). $$

The six terms are easily interpretable. For example, Cw-Cm reflects whether there is a stronger tendency for women than for men to have their first child with a person who already had a child. One could, in principle, decompose differently without going beyond demographic variables, by combining some of these terms or splitting them into smaller components, but this would only be meaningful if it provides better substantive insight.

The difference Bw-Bm deserves elaboration: Bw is the number of first births women in certain cohorts (e.g. 1956–1958) have had with men in any cohort who have no older children, divided by the number of women in these cohorts (ignoring other restrictions now for simplicity). Similarly, Bm is the number of first births men in two year older cohorts (e.g. 1954–1956) have had with women in any cohort who have no older children, divided by the number of men in these cohorts. In the hypothetical situation where women and men have all births in this category with partners who are two years older or younger, respectively, the two numerators are equal. In that case, Bw and Bm would only differ if the sizes of these female and male birth cohorts among Norwegian residents at age 45 are different. In reality, women and men also have first births with partners outside these cohorts, but it turns out that this matters much less than the difference between the sizes of the male and female cohorts—referred to below as relative cohort size. This argument, which may be seen as involving a decomposition of Bw-Bm, is described in more detail in the Supplementary Material.

The relative cohort size also influences the other differences, such as Cw-Cm, but far less—especially in the absolute term. In other words, Bw-Bm reflects to a large extent relative cohort size, and relative cohort size affects the sex gap in childlessness largely through the B-component. This also is explained in more detail in the Supplementary Material.

The relative cohort size among those living in Norway at age 45 is in turn a result of the development in the number of births over the respective years (e.g. from 1954–1956 to 1956–1958), sex ratios at birth, and mortality, emigration, and immigration patterns. However, the *change* in the relative cohort size from the older cohort group to the younger turns out to be driven almost entirely by the fertility trends. This is elaborated on in the Supplementary Material and notes 8 and 9 to the Results section.

The procedure is slightly different when distinction is made between Norwegian-born and immigrants. When the Norwegian-born are considered, Bw, Bm, Cw, and Cm refer to births where the other parent is also a Norwegian-born, lived in Norway at age 45, was not older than 45 at birth, and either had no older children (B) or had older children (C). The proportions Aw1, Aw2, Am1, and Am2 refer to births where the other parent was an immigrant, lived in Norway at age 45, was not older than 45 at birth, and either had not older children (1) or had older children (2). Dw4 and Dm4 refer, as before, to births with an unidentified co-parent, and Dw1, Dw2, and Dw3 (for simplicity combined into Dw123 for women and Dm12 for men) are also defined as above, i.e. without regard to the other parent’s earlier childbearing or country of birth). The procedure is the same for immigrants, except that “Norwegian-born” is substituted with “immigrant”, and vice versa.

### The Norwegian Data

The Norwegian Population Register includes all persons who have lived in Norway after 1964 and provides information about, for example, country of birth, sex, and years of birth, death, immigration, and emigration. It also includes a personal identification number (PIN) for each person, and for almost everyone born in Norway after 1953 there are PINs of mothers and fathers. This means that there are almost complete birth histories for women and men born after 1935, who must have had almost all their children after 1953. However, some children born in Norway after 1953 are registered with only a mother, only a father, or even no parent. For example, the father may have a PIN, but not be reported by the mother, or the father may never had lived in Norway and thus not have a PIN. The latter is particularly likely among immigrants, who may have arrived in the country with only the mother. A quite different issue is that some adult immigrants—probably men in particular—may have children they have not brought with them to Norway and who are therefore not included in the register and will not be counted at all in an analysis based on that data source. The version of these data that were available for the study covers the period up to 2018.

## Results

### The Cohorts from the Mid-1950s

Among women born 1956–1958 who lived in Norway at age 45, a proportion of 0.886 (Sw) became mothers, i.e. 11.4% remained childless as shown in Table 1. The proportion having a first child with a man who had no older children, lived in Norway at age 45, and was no older than 45 at the time of birth (Bw) was 0.748. (For simplicity, when “women” or “men” is written below, it refers to residents in Norway at age 45 unless otherwise specified.) The women had their remaining first children with men who were not previously childless (Cw = 0.070), childless men (Dw1 = 0.027) or fathers (Dw2 = 0.003) who did not live in Norway at age 45, men who were older than 45 (Dw3 = 0.009), or men who were not identified (Dw4 = 0.028). In other words, many of the women had a first birth without also bringing a man not older than 45 and living in Norway at age 45 out of childlessness.

**Table 1:** Contributions to proportion parents among women and men

	The other parent was 45 or younger at time of birth				The other parent was older than 45 at time of birth	The other parent was unidentified	Total
	The other parent lived in Norway at age 45		The other parent did not live in Norway at age 45				
	The other parent had no older children	The other parent had older children	The other parent had no older children	The other parent had older children			
First births among women born 1971–1973 (Nw = 109,808)	Bw = 0.700	Cw = 0.084	Dw1 = 0.021	Dw2 = 0.002	Dw3 = 0.018	Dw4 = 0.044	Sw = 0.869
First births among men born 1969–1971 (Nm2 = 117,299)	Bm = 0.654	Cm = 0.092	Dm1 = 0.013	Dm2 = 0.001		Dm4 = 0.006	Sm = 0.767
Difference 1	0.046	−0.008	0.008	0.001	0.018	0.038	0.102
First births among women born 1956–1958 (Nw = 94,212)	Bw = 0.748	Cw = 0.070	Dw1 = 0.027	Dw2 = 0.003	Dw3 = 0.009	Dw4 = 0.028	Sw = 0.886
First births among men born 1954–1956 (Nm2 = 95,900)	Bm = 0.742	Cm = 0.065	Dm1 = 0.016	Dm2 = 0.002		Dm4 = 0.003	Sm = 0.828
Difference 2	0.006	0.005	0.011	0.001	0.009	0.025	0.058
Difference 1 – Difference 2	0.040	−0.013	−0.003	0.000	0.009	0.013	0.044

The “Total” may differ from the sum of the other numbers in the row above because of rounding. “Lived in Norway at age 45” means “lived in Norway at the end of the year when they turned 45 or, if born after 1973, the end of 2018”

Turning to men in the 1954–1956 cohorts, almost the same proportion had their first child with a co-parent who had no older children and lived in Norway at age 45 (Bm = 0.742). The contribution to men’s exit from childlessness before age 45 from women’s second- or higher-order children was Cm = 0.065, and the contributions from previously childless women or mothers who did not live in Norway at age 45 were Dm1 = 0.016 and Dm2 = 0.002. Additionally, 0.3% of the men in the 1954–1956 cohorts had a first child with an unidentified woman (Dm4 = 0.003). This sums up to 0.828 (Sm), i.e. a childlessness of 17.2%.

Thus, the difference between women’s and men’s childlessness, which is 5.8 percentage points (Sw-Sm = 0.058), is partly a result of a larger proportion of women whose co-parent is unidentified, because of not having been reported or never having lived in Norway (Dw4-Dm4 = 0.025). There is also a larger proportion of women than men who had their first child with a childless co-parent not living in Norway at age 45 (Dw1-Dm1 = 0.011), and some women had their first child with a man older than 45 (Dw3 = 0.009). The other contributions to the sex difference are smaller. Most interestingly perhaps, it was only slightly more common for childless women than childless men to have a child with a partner who already had a child (Cw-Cm = 0.005), and the contributions from those who had their first child with a co-parent who was also childless are quite similar as well (Bw-Bm = 0.006).^7^

### The younger cohort group

Among women born in 1971–1973, the proportion who became mothers was 0.869, i.e. 13.1% childlessness. The contributions that sum up to 0.869 are Bw = 0.700, Cw = 0.084, Dw1 = 0.021, Dw2 = 0.002, Dw3 = 0.018, and Dw4 = 0.044. The corresponding proportions for men born in 1969–1971 are Sw = 0.767 (i.e. 23.3% childlessness), Bm = 0.654, Cm = 0.092, Dm1 = 0.013, Dm2 = 0.001, and Dm4 = 0.006.

The main reason for the 4.4 percentage points larger sex difference in childlessness in the younger cohort group than in the older is that the difference between Bw and Bm is much larger: 0.046 in the younger cohort group, as opposed to only 0.006 in the older. This is largely because the relative cohort size (ratio between number of men born two years earlier and number of women) among residents in Norway at age 45 is much higher: 1.068 in the younger cohort group as opposed to 1.018 in the older.^8^ This pattern in turn reflects the fertility decline that started in the mid-1960s (Kravdal, 2016).^9^

In the youngest cohort group, there was also a larger proportion of women who had their first child with a man older than 45 (Dw3 = 0.018) than in the older cohorts (Dw3 = 0.009), and there was a larger sex difference in the proportion having an unidentified co-parent (Dw4-Dm4 going up from 0.025 to 0.038).

However, there are also two weaker trends in the opposite direction, the most important being the change in the C-component. Both Cw and Cm, which refer to first-born children with a co-parent who already had a child (i.e. who has re-partnered), were higher in the younger cohort group than in the older, but this increase was especially pronounced for men: While there was more “recycling” of men than women in the older cohort group (Cw-Cm positive), the opposite was the case in the younger (Cw-Cm negative). The second contribution in this direction is the small decline in the tendency that women to a larger extent than men have their first child with a previously childless person not living in Norway at age 45 (Dw1-Dm1 has gone down by 0.003).

To summarize, the 4.4 percentage point increase in the sex gap in childlessness over 15 years is to a large extent a result of the B-component, which reflects the fertility development during earlier decades. Additionally, there have been some smaller changes that have almost counteracted each other.

### A Distinction between Norwegian-Born and Immigrants

**Fig. 2:**
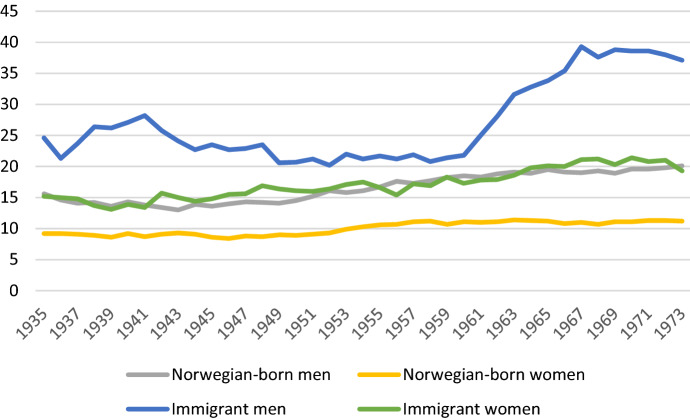
Percent childless at age 45, by sex, country of birth, and birth cohort, in Norway. *Note*: Calculated by the author; see text for description of data

If the population is divided into Norwegian-born and immigrants, two quite different pictures emerge. Among Norwegian-born women, the proportion childless has increased very little, from 11.0% in the 1956–1958 cohorts to 11.2% in the 1971–1973 cohorts (see Fig. 2 for a description for one-year cohorts). Among Norwegian-born men born two years earlier, the increase was from 16.8% to 19.4%. In other words, the sex gap in childlessness increased by 2.4 percentage points, from 5.8 to 8.2.

The proportion childless has been consistently higher for both sexes in the (steadily growing) immigrant population and also increased more. Among women, the childlessness increased from 16.4% to 20.3% over the 15-year period, while it increased even more among men, from 21.3% to 38.7%. Thus, the gap between the sexes increased from 4.9 to 18.4 percentage points (i.e. more than a tripling). As mentioned, however, it is possible that especially immigrants may have children who are not included in the population register—because they have never lived in Norway—and who therefore contribute neither to men’s nor women’s calculated fertility. In particular, immigrants who have arrived at a relatively high age may have children in their home country whom they have not brought with them. This is a particularly relevant concern for men; it is less likely that women immigrate without their children.^10^

The decomposition results are shown in Table 2. Starting with the oldest cohort group and a focus on the Norwegian-born, 73.8% of the women had a first child with a previously childless Norwegian-born man no older than 45 (Bw = 0.738). The corresponding number for men (Bm) is 0.724. The proportion of Norwegian-born women who had a first child with a Norwegian-born man who already had children (Cw = 0.068) is only slightly larger than the corresponding proportion for Norwegian-born men (Cm = 0.063). Furthermore, it was slightly more common for Norwegian-born women than Norwegian-born men to have a first child with an immigrant partner (Aw1 + Aw2 = 0.031 vs Am1 + Am2 = 0.029). Finally, there is one more factor that contributes to make more Norwegian-born women than men parents: 5.3% of the women had a first child with a man not living in the country at age 45 or older than 45 at birth (Dw123 = 0.035) or who was not identified (Dw4 = 0.018), while the corresponding proportion among Norwegian-born men was 1.5% (Dm12 = 0.015 and Dm4 = 0.000). Thus, the 5.8 percentage points higher proportion parents among Norwegian-born women than Norwegian-born men is to a large extent a result of the D-components, while B contributes less than half of that and the other components much less.

**Table 2:** Contributions to proportion parents among women and men

Norwegian-born							
	The other parent was 45 or younger at time of birth and lived in Norway at age 45				The other parent was older than 45 at time of birth or did not live in Norway at age 45	The other parent was unidentified	Total
	The other parent was born in Norway		The other parent was immigrant				
	The other parent had no older children	The other parent had older children	The other parent had no older children	The other parent had older children			
First births among women born 1971–1973 (Nw = 87,526)	Bw = 0.700	Cw = 0.085	Aw1 = 0.048	Aw2 = 0.006	Dw123 = 0.031	Dw4 = 0.018	Sw = 0.888
First births among men born 1969–1971 (Nm2 = 93,667)	Bm = 0.642	Cm = 0.096	Am1 = 0.050	Am2 = 0.008	Dm12 = 0.009	Dm4 = 0.001	Sm = 0.806
Difference 1	0.058	−0.011	−0.002	−0.002	0.022	0.017	0.082
First births among women born 1956–1958 (Nw = 85,948)	Bw = 0.738	Cw = 0.068	Aw1 = 0.027	Aw2 = 0.004	Dw123 = 0.035	Dw4 = 0.018	Sw = 0.890
First births among men born 1954–1956 (Nm2 = 88,068)	Bm = 0.724	Cm = 0.063	Am1 = 0.026	Am2 = 0.003	Dm12 = 0.015	Dm4 = 0.000	Sm = 0.832
Difference 2	0.014	0.005	0.001	0.001	0.020	0.018	0.058
Difference 1 – Difference 2	0.044	−0.016	−0.003	−0.003	0.002	−0.001	0.024

Immigrants							
	The other parent was 45 or younger at time of birth and lived in Norway at age 45				The other parent was older than 45 at time of birth or did not live in Norway at age 45	The other parent was unidentified	Total
	The other parent was immigrant		The other parent was born in Norway				
	The other parent had no older children	The other parent had older children	The other parent had no older children	The other parent had older children			
First births among women born 1971–1973 (Nw = 22,282)	Bw = 0.326	Cw = 0.025	Aw1 = 0.185	Aw2 = 0.031	Dw123 = 0.084	Dw4 = 0.146	Sw = 0.797
First births among men born 1969–1971 (Nm2 = 23,632)	Bm = 0.341	Cm = 0.023	Am1 = 0.165	Am2 = 0.025	Dm12 = 0.034	Dm4 = 0.026	Sm = 0.613
Difference 1	−0.015	0.002	0.020	0.006	0.050	0.120	0.184
First births among women born 1956–1958 (Nw=8264)	Bw = 0.315	Cw = 0.018	Aw1 = 0.258	Aw2 = 0.038	Dw123 = 0.080	Dw4 = 0.128	Sw = 0.836
First births among men born 1954–1956(Nm2 = 7,832)	Bm = 0.371	Cm = 0.016	Am1 = 0.278	Am2 = 0.042	Dm12 = 0.052	Dm4 = 0.028	Sm = 0.787
Difference 2	−0.056	0.002	−0.020	−0.004	0.028	0.100	0.049
Difference 1 – Difference 2	0.041	0.000	0.040	0.010	0.022	0.020	0.135

The “Total” may differ from the sum of the other numbers in the row above because of rounding. “Lived in Norway at age 45” means “lived in Norway at the end of the year when they turned 45 or, if born after 1973, the end of 2018”

Bw-Bm is considerable larger in the younger than in the older cohort group (0.058 versus 0.014) and is essentially the only component that contributes to the (moderately) increasing sex gap in childlessness among the Norwegian-born. The A-component, which contributes very little to the sex difference in childlessness in the older cohort group, contributes little also in the younger, and actually in the opposite direction. Furthermore, while the D-components contribute much to the sex difference in childlessness in the older cohort group, the contributions in the younger cohort group are equally large. However, and as also observed when Norwegian-born and immigrants were pooled together (Sect. 3.2), the change in the C-component runs counter to that in the B-component, in addition to being weaker: While there was some more “recycling” of men than women among Norwegian-born in the older cohort group (Cw-Cm positive), the opposite was the case in the younger cohort group (Cw-Cm negative).

More detailed examination shows that about two-thirds of the change in Bw-Bm over time is a result of women in the younger cohort group being more clearly outnumbered by the two year older men than women in the older cohort group (as a reflection of fertility trends some decades ago).^11^ This change in the relative cohort size contributes much less to the increasing sex gap in childlessness through the A-, C-, and D-components.^12^

Based on similar calculations for immigrants, the following can be concluded: Immigrant men’s childlessness is more different from immigrant women’s childlessness in the younger cohort group than in the older partly because of the B-component, which largely reflects a larger number of immigrant men compared to women in the younger cohort group (the increase in relative cohort size is actually stronger than among the Norwegian-born). Additionally, while it was less common for immigrant women to have their first child with a Norwegian-born man than it was for immigrant men to have their first child with a Norwegian-born woman in the older cohort group (Aw1 + Aw2-Am1-Am2 negative), the situation was opposite in the older cohort group (Aw1 + Aw2-Am1-Am2 positive). In other words, there has been a particularly large decline in immigrant men’s (compared to immigrant women’s) tendency to have a first child with a person born in Norway. The third contribution, which has about the same size as the other two, comes from the D-components. Most importantly, it has become more common for immigrant women to have an unidentified co-parent (Dw4 increasing from 0.128 to 0.146), and it has become less common for immigrant men to have a child with a woman who did not live in Norway at age 45 (the remaining D-component going down from 0.052 to 0.034).

## Summary and Conclusion

According to data from the population register (on which also Norway’s official population statistics is based), the sex difference in childlessness within the subgroup born in Norway has increased moderately over the last 15 years, from 5.8 to 8.2 percentage points (i.e. by 2.4). This is almost entirely a result of a growing difference between the proportion of Norwegian-born women who had their first child with a Norwegian-born person who had no older children (and was younger than 45 at the time of birth and lived in Norway at age 45) and the corresponding proportion among Norwegian-born men. The main factor behind this is the increase in the number of men in the two year older cohorts relative to the number of women, which primarily reflects the fertility trends from the mid-1950s to the mid-1970s (mortality and emigration patterns do not matter.) One might expect that the widening sex gap in childlessness could be partly due to changing sex asymmetries in re-partnership, but that is not the case. On the contrary, women’s inclination to have their first child with a man who already has a child has increased *less* than men’s inclination to have a first child with a woman who already has a child. In principle, a sex difference in childlessness can arise also because more women than men have a first child with an immigrant, with a person who does not live in Norway at age 45 (when fertility is measured), with a person older than 45, or with an unidentified person. However, there has not been a change in these patterns among the Norwegian-born over the study period. To summarize, the widening of the sex gap can to a large extent be seen as some sort of “demographic necessity”, produced by earlier fertility trends. It is not a result of an increasing acceptance of having step-children (and thus perhaps a relatively old partner) especially among women, or another change that could lead to a particularly large increase in the proportion of women having a first child with a person who is already a parent. This has implications for future research, because if there had been a development in such a direction, a reasonable next step would have been to seek social explanations for it.

Among immigrants, the sex difference in childlessness has increased much more, from 4.9 to 18.4 percentage points. There are four main contributions to this increase (and one that is smaller, while nothing has offsetting impact): First, the contribution that drives the moderate widening of the sex gap in childlessness among Norwegian-born has almost the same size for immigrants, and reflects an increase in the number of immigrant men compared to women. Second, there has been a particularly large decline in immigrant men’s (compared to immigrant women’s) tendency to have a first child with a Norwegian-born person. Third, it has become less common for immigrant men to have a child with a woman who did not live in Norway at age 45, and fourth, it has become more common for immigrant women to have an unidentified co-parent. In some cases where the woman had a child with an unidentified father, the father may actually be included in the register, but not reported by the mother. Some of these unreported fathers may be immigrants, although that is not necessarily more likely than that they are Norwegian-born. In other words, the fourth contribution to the increasing sex gap in childlessness among immigrants, which is not large, may in fact be even smaller. Anyway, even if the contribution is blown up because of this type of underreporting, it may be reasonable enough if we instead apply a more “social” definition of fertility, because if immigrant men have actually fathered such children who are not registered with a father—and perhaps to an increasing extent over the years—they have likely had very little contact with these children.

It should be noted, however, that this is an analysis of registered births. It is possible that immigrants have children they have not brought with them to Norway and who are therefore not included in the register (and thus contribute to neither women’s nor men’s calculated fertility). Such a situation is particularly likely among male immigrants, in which case the real sex difference in childlessness is smaller than suggested by the analysis presented here.

As mentioned, this decomposition approach may also be used when analysing other population groups than Norwegian-born and immigrants, for example, various educational categories. Although women’s education is one of the most commonly studied determinants of fertility (Beaujouan et al., 2016; Rybniska 2020; Woods et al. 2014), and this research has been extended to men in recent years (Jalovaara et al., 2019; Kravdal & Rindfuss, 2008), there has not been an explicit interest in the education-specific sex gap in childlessness. In a decomposition along such lines, one should, of course, take into account whether the co-parent is in the same or a different educational category.

Although register data have been used in this study, the same decomposition can be done, and would be meaningful, with surveys based on a randomly drawn sample of women and men who are resident in a certain country—provided that the data include the same kind of information on both the index person and the co-parent. Only the component related to the sex difference in the tendency to not report the other parent would have to be modified, so it instead reflects how common it is that the co-parent has characteristics not adequately reported. One may be concerned about survey respondents possibly not reporting all their own births (which is another issue than inadequate information about the co-parent), and perhaps the men in particular, but as mentioned there may be a corresponding problem with immigrants when register data are used.

## Supplementary Information

Below is the link to the electronic supplementary material.

Supplementary file1 (DOCX 33 kb)

## Data Availability

The use of the data for the purpose of this study is approved by the Regional Committees for Medical and Health Research Ethics (2018/434) and the data owners. The data are strictly protected and only available through collaboration with the Centre for Fertility and Health.

## Footnotes

1 There are many pathways to childlessness. For example, some people remain childless because they have never lived in a relationship they consider as stable enough for parenthood (Hart 2019). Others may have had a partner for a quite long time, but not wanted a child. Alternatively, they may have wanted to postpone childbearing until a later age, when it turned out that they were no longer able to have a child, or they may have been infecund at least since the start of their relationship.

2 The increase in childlessness is probably to a large extent a result of social, ideational and other changes that have led to less stable partnerships, weakened the interest in childbearing generally, or at least induced people to want a late entry into parenthood, and made it easier to avoid unwanted births. These changes include a longer period spent in school (Ní Bhrolcháin and Beaujouan 2012), increasing economic uncertainty among young people (in some countries), new contraceptive technologies, improved access to abortion, and possibly a stronger preference for activities that compete with family life (Lesthaeghe 2014). Furthermore, some evidence suggests that men’s age-specific ability to conceive may have gone down because of reduced semen quality (Levine et al. 2017).

3 Some individual-level adverse consequences may, of course, be expected by the decision-makers, who may have concluded that they nevertheless prefer to remain childless because the benefits more than compensate. In that case, and especially if the decision is not regretted, the consequences should perhaps not be seen as a “problem” that deserves public attention (Kravdal 2019).

4 The impact that childlessness has on individuals’ lives may have further implications for society. This may be referred to as a (quasi) aggregate-level effect. Additionally, a high proportion childless is typically linked to generally low fertility, which may cause population decline and ageing. The latter has often been considered as challenging for the country’s welfare system and potentially pushing the economic growth down (Bonenkamp et al. 2017; de Meijer et al. 2013; Rechel et al. 2013). On the other hand, it can also be argued that there may be economic and environmental *advantages* from having a population that is older and grows less rapidly (Lee and Mason 2010; McDonald et al. 2006; Weber and Sciubba 2019).

5 For example, among men born in 1961–63 (who can be observed up to age 55), the proportion childless fell by only 0.9 percentage point from age 45 to 50 and 0.2 percentage point from age 50 to 55.

6 To the extent that there are causal effects, these are probably partly a result of a less healthy lifestyle among the childless and a lower availability of practical and emotional support in old age (Brandt et al. 2009; Wenger et al. 2007). Additionally, there are certain biological effects of pregnancy among women (Husby et al. 2018; Troisi et al. 2018).

7 As mentioned in the Supplementary Material, the number of births of this type is somewhat larger for men than women (F^(a)^1* is 1.0% larger than F1, i.e. (F1- F^(a)^1*)/ F^(a)^1* =  −0.010). However, there is also a larger male cohort (Nm2 is 1.8% larger than Nw, i.e. Nm2/Nw = 1.018 and 1-Nm2/Nw =  −0.018). The latter dominates, so Bw is 0.8% (=  −0.010-( −0.018)) larger than Bm. In absolute numbers, this is 0.006 (= 0.008*Bm).

8 The number of births in the relevant category is only 0.1% smaller for men than for women in the younger cohort group (i.e. F^(a)^1* is 0.1% smaller than F1, using the terminology in the Supplementary Material), while there is a 1.0% difference in the opposite direction in the older cohort group. However, that does not contribute much to the change in Bw-Bm. In fact, in the absence of this change, the increase in Bw-Bm over time would have been 0.033 instead of 0.040.

9 The increasing male surplus (from 1.018 to 1.068) is primary driven by the middle term in Eq. (5) in the Supplementary Material, i.e. the extent to which cohort sizes have increased over two-year periods (from 1954–1956 to 1956–1958 in the older cohort group, and from 1969–1971 to 1971–1973 in the younger cohort group). This term has increased from 0.998 to 1.036. All of this is a result of the patterns in the number of births, because if it is not conditioned on residence in Norway at age 45, the term increases from 0.998 to 1.037. The development in deaths and emigration contribute nothing. The sex ratio among the resident women and men (the first term in Eq. 5) has only increased from 1.026 to 1.033. The sex ratio at birth has increased more than this, from 1.047 to 1.059, but the developments in deaths and emigration have contributed in the opposite direction. The immigration component (the third term in Eq. 5), has changed very little, from 0.994 to 0.998.

10 There is much variation in childlessness within the immigrant population (not shown in figures or tables). For example, within the youngest cohort group, the proportion childless was 49% among men from non-Nordic European countries (even though only 11% were never-married with no cohabitation experience after 2005), while it was 30% among African men and 20% among Asian men. The highest figures, almost 70%, are seen among men from the Baltic countries and Poland. Childlessness was generally most common among those arriving after age 30. Given these patterns and levels, it seems indeed plausible that the actual childlessness is lower than what the register data suggest.

11 The change in Bw-Bm arises from two changes that work in the same direction: First, the number of births in the numerator in the Bw ratio (F1 according to the notation in the Supplementary Material) is more markedly larger than that in the Bm ratio (F^(a)^1*) in the younger cohort group than in the older cohort group. Second, the women in the younger cohort group are more clearly outnumbered by the two year older men. This second contribution is twice as large as the first.

12 In particular, if the relative cohort size had been the same in the younger cohorts as in the older ones, rather than larger, Cw-Cm would—everything else unchanged—have increased by  −0.020 instead of  −0.016. Thus, one can say that the contribution to the increasing sex gap in childlessness that comes from the C-component, which is  −0.016, is the sum of two parts: 0.004 from the change in the relative cohort size, and  −0.020 from the remaining factors, which may be described as changes in the tendencies for women and men to have their first child with a partner who already is a parent. There is even less impact of the change in the relative cohort size through the A- and D-components, since these components are generally smaller.

## Code availability

The code has no value without access to the data.

## References

[CR1] Beaujouan E, Brzozowska Z, Zeman K (2016). The limited effect of increasing educational attainment on childlessness trends in twentieth-century Europe, women born 1916–65. Population Studies.

[CR25] Bonenkamp J, Meijdam L, Ponds E, Westerhout E (2017). Ageing-driven pension reforms. Journal of Population Economics.

[CR31] Brandt M, Haberkern K, Szydlik M (2009). Intergenerational help and care in Europe. European Sociological Review.

[CR26] de Meijer C, Wouterse B, Polder J, Koopmanschap M (2013). The effect of population aging on health expenditure growth: A critical review. European Journal of Ageing.

[CR2] Dribe M, Nystedt P (2017). Age homogamy, gender, and earnings: Sweden 1990–2009. Social Forces.

[CR3] Frejka T, Kreyenfeld M, Konietzka D (2017). Childlessness in the United States. Childlessness in Europe: Contexts, causes, and consequences.

[CR4] Grasdal AL, Lommerud KE (2019). Barnløshet blant men i Norge – hvem er de, og hvor bor de?. Tidsskrift for Velferdsforskning.

[CR5] Guilmoto CZ (2009). The sex ratio transition in Asia. Population and Development Review.

[CR20] Hart RK (2019). Union histories of dissolution: What can they say about childlessness?. European Journal of Population.

[CR33] Husby A, Wohlfahrt J, Øyen N, Melbye M (2018). Pregnancy duration and breast cancer risk. Nature Communications.

[CR6] Jalovaara M, Neyer G, Andersson G, Dahlberg J, Dommermuth L, Fallesen P, Lappegård T (2019). Education, gender, and cohort fertility in the Nordic countries. European Journal of Population.

[CR7] Kravdal Ø, Rindfuss RR, Choe MK (2016). Not so low fertility in Norway – a result of affluence, liberal values, gender-equality ideals, and the welfare state. Low fertility, institutions, and their policies: Variations across industrialized countries.

[CR24] Kravdal Ø (2019). What kind of individual-level effects of childbearing would we ideally be interested in learning about? The important distinction between expected, unexpected, varying and general effects. Journal of Population Research.

[CR8] Kravdal Ø, Rindfuss RR (2008). Changing relationships between education and fertility – a study of women and men born 1940–64. American Sociological Review.

[CR9] Kravdal Ø, Tverdal Aa, Grundy E (2020). The association between parity, CVD mortality, and CVD risk factors among Norwegian women and men. European Journal of Public Health.

[CR10] Lappegård T, Rønsen M, Skrede K (2011). Fatherhood and fertility. Fathering.

[CR28] Lee R, Mason A (2010). Fertility, human capital, and economic growth over the demographic transition. European Journal of Population.

[CR22] Lesthaeghe R (2014). The second demographic transition: A concise overview of its development. Proceedings of the National Academy of Sciences.

[CR23] Levine H, Jørgensen M, Martino-Andrade A, Mendiola J, Weksler-Derri D, Mindlis I (2017). Temporal trends in sperm count: A systematic review and meta-regression analysis. Human Reproduction Update.

[CR29] McDonald GW, Forgie VE, MacGregor C (2006). Treading lightly: The ecofootprints of New Zealand’s ageing population. Ecological Economics.

[CR11] Ní Bhrolcháin M (2005). The age difference at marriage in England and Wales: A century of patterns and trends. Population Trends.

[CR21] Ní Bhrolcháin M, Beaujouan E (2012). Fertility postponement is largely due to rising educational enrolment. Population Studies.

[CR27] Rechel B, Grundy E, Robine JM, Cylus J, Mackenbach JP, Knai C, McKee M (2013). Ageing in the European Union. The Lancet.

[CR13] Rybińska A (2020). A research note on the convergence of childlessness rates between women with secondary and tertiary education in the United States. European Journal of Population.

[CR14] Schmitt C (2021). The impact of economic uncertainty, precarious employment, and risk attitudes on the transition to parenthood. Advances in Life Course Research.

[CR15] Schoumaker B (2019). Male fertility around the world and over time: How different is it from female fertility. Population and Development Review.

[CR16] Sobotka T, Kreyenfeld M, Konietzka D (2017). Childlessness in Europe: Reconstructing long-term trends among women born in 1900–1972. Childlessness in Europe: Contexts, causes, and consequences.

[CR12] Statistisk Norway (2021). Number of births, by age and cohort (Table 3). Online at www.ssb.no/en/befolkning/statistikker/fodte

[CR34] Troisi R, Bjørge T, Gissler M, Grotmol T, Kitahara CM, Myrtveit Saether SM (2018). The role of pregnancy, perinatal factors and hormones in maternal cancer risk: A review of the evidence. Journal of Internal Medicine.

[CR30] Weber H, Sciubba JD (2019). The effect of population growth on the environment: Evidence from European regions. European Journal of Population.

[CR32] Wenger GC, Dykstra PA, Melkas T, Knipscheer KCM (2007). Social embeddedness and late-life parenthood – community activity, close ties, and support networks. Journal of Family Issues.

[CR17] Wood J, Neels K, Kil T (2014). The educational gradient of childlessness and cohort parity progression in 14 low fertility countries. Demographic Research.

