# Supplementary material for: Sex Differences in Childlessness in Norway: Identification of Underlying Demographic Drivers
Source: Eur J Popul. 2021 Jul 23;37(4-5):1023–41. doi: 10.1007/s10680-021-09590-4 (PMC8575743; doi:10.1007/s10680-021-09590-4)
Supplement: Supplementary file 1 — Supplementary file1 (DOCX 33 kb) [file 10680_2021_9590_MOESM1_ESM.docx]

**SUPPLEMENTARY MATERIAL**

**More detailed specification of the decomposition and discussion of its components**

Let us use the oldest cohort group (1954-56 for men and 1956-58 for women) as an example and start with the following definitions:

Nw: Number of women born 1956-58 and living in Norway at age 45

F: Number of first births to these women

H: Number of higher-order births to these women

Fx: Number of first births to women born 1956-58 and *not* living in Norway at age 45

Hx: Number of higher-order births to women born 1956-58 and *not* living in Norway at

age 45

(The expression “living in Norway at age 45” means “living in Norway at the end of the year when they turned 45 or, if born after 1973, the end of 2018”.) Twins and higher-order multiple births count as only one birth when calculating these numbers. This is because when, for example, a woman gives birth to twins, it is only one man who – if he does not already have children - becomes a father.

Each of F, H, Fx, and Hx can be divided into six categories F1-F6, H1-H6, Fx1-Fx6, and Hx1-Hx6 according to the following characteristics of the father at the time of the birth or at age 45:

1: Father was 45 or younger at birth, lived in Norway at age 45, had no older children

2: Father was 45 or younger at birth, lived in Norway at age 45, had older children

3: Father was 45 or younger at birth, did not live in Norway at age 45, had no older children

4: Father was 45 or younger at birth, did not live in Norway at age 45, had older children

5: Father was older than 45 at birth (without regard to residence or earlier childbearing)

6: Father was not identified

This partition of births into 4*6 categories is illustrated in the upper part of Supplementary Table S1, which includes the mentioned symbols and (to make the presentation more concrete) numbers.

The proportion of women living in Norway at age 45 who have become mothers is, of course, Sw = F/Nw, and the six contributions to this ratio are:

Bw = F1/Nw

Cw = F2/Nw

Dw1 = F3/Nw

Dw2 = F4/Nw

Dw3 = F5/Nw

Dw4 = F6/Nw.

Thus, the proportion Bw refers to first births to women which are also first births to men younger than 45 and living in the country at age 45, and Cw refers to first births to women which are second or higher-order births to men younger than 45 and living in the country at age 45. Dw1-Dw4 refer to the remaining categories of women’s first births.

Men who lived in Norway at age 45 have not only had a first child before that age because of births such F1; births such as H1, Fx1 and Hx1 come in addition. One might consider assuming, for simplicity, that women born in 1956-58 have all their children with men born in 1954-56. Under that assumption the proportion who become fathers among the Nm2 men in those cohorts who lived in Norway at age 45 would be

F1/Nm2 + H1/Nm2 + Fx1/Nm2 + Hx1/Nm2

plus a small proportion who had their first child with an unidentified mother - a detail that is further discussed below. (Nm2 is chosen as the symbol instead of Nm because the men are born two years before the Nw women that are considered. While this terminology is not helpful here, it facilitates a later argument.)

In reality, all the births F1, H1, Fx1 and Hx1 are, of course, not with men in the 1954-56 cohorts; it is about 40% that are with such men, and let us denote these as F1*, H1*, Fx1* and Hx1* (see illustration in lower part of Supplementary Table S1). The births in category 1 among women in other cohorts than the 1956-58 cohorts can be denoted as F^(o)^1, H^(o)^1, Fx^(o)^1 and Hx^(o)^1, and among these, F^(o)^1*, H^(o)^1*, Fx^(o)^1* and Hx^(o)^1* are with men born 1954-56. The sums of the numbers for the female 1956-58 cohorts and those for the other female cohorts, i.e. all cohorts of women, are referred to as F^(a)^1, H^(a)^1, Fx^(a)^1, Hx^(a)^1, F^(a)^1*, H^(a)^1*, Fx^(a)^1* and Hx^(a)^1*. It actually turns out that F^(a)^1*, H^(a)^1*, Fx^(a)^1* and Hx^(a)^1* are not very different from F1, H1, Fx1 and Hx1. In other words, the assumption mentioned above about all women having their children with two year older men would have given an almost correct picture of the proportion childless men in the 1954-56 cohort. Nevertheless, in the decomposition analysis that is done, all births regardless of the mother’s cohort are taken into account when calculating men’s childlessness and the contributions to this – just as all births regardless of the father’s cohort are taken into account when calculating Sw, Bw, Cw and Dw1-Dw4.

Some men in the 1954-56 cohorts have their first child with a woman who is not identified and therefore, of course, not among those contributing to the F^(a)^1*, H^(a)^1*, Fx^(a)^1* and Hx^(a)^1*. If we refer to the number of such births as U, the total number of first births to the men in the 1954-56 cohorts is

S = F^(a)^1* + H^(a)^1* + Fx^(a)^1* + Hx^(a)^1* + U,

and if each of these terms is divided by Nm2, we get the following proportion Sm who become fathers:

Sm = Bm + Cm + Dm1 + Dm2 + Dm4.

The symbols Bm, Cm, Dm1, Dm2 and Dm4 are chosen because they refer to categories of first births that, from the man’s perspective, are the same as those referred to by Bw, Cw, Dw1, Dw2 and Dw4. For example, Cm is the proportion of men who have, so to speak, been brought into parenthood by a woman who already had a child and who lived in Norway at age 45, while Cw is the proportion of women who have been brought into parenthood by a man who was then no older than 45, already had a child, and lived in Norway at age 45. Note that there is no Dm3 term. That is because, while women can have children with men older than 45 (category 5, which is the basis for the definition of Dw3), it is extremely uncommon for women to have children at that age. In total, among all the births F^(a)^1* + H^(a)^1* + Fx^(a)^1* + Hx^(a)^1*, only 6 are among women older than 45, and these are not taken out as a separate category.

The difference between the proportion of women who are mothers and the proportion of men who are fathers can be written as

1. Sw-Sm = (Bw-Bm) + (Cw-Cm) + (Dw1-Dm1) + (Dw2-Dm2) + Dw3 + (Dw4-Dm4).

The difference Bw-Bm deserves elaboration, which can be done through further decomposition: Since Bw=F1/Nw and Bm=F^(a)^1*/Nm2, the following is a reasonable approximation when there are small differences between F1 and F^(a)^1 and between Nw and Nm2:

1. Bw-Bm ≈ (F1-F^(a)^1*)/Nm2 – (Nw-Nm2) F^(a)^1*/Nm2^2^.

(Generally, when y=x/z, the change Δy in y as a function of the changes in x and z is

Δy = (Δx∙z_av_ - Δz∙ x_av_)/((z_av_-Δz/2)(z_av_+Δz/2)),

where x_av_ and z_av_ are the averages between the start and end values of these two variables. They can be approximated by the start or end values when Δx and Δz are small.)

The relative difference between the Bs is

1. (Bw-Bm)/Bm ≈ (F1-F^(a)^1*)/ F^(a)^1* – (Nw-Nm2) /Nm2.

In other words, in a situation where F1 and F^(a)^1* are equal, the relative difference between Bw and Bm is the same as the relative differences between the size of the female cohorts and the two year older male cohorts (among those resident at age 45). Given the assumption about relatively small differences, the second term of (3) can alternatively be written as (Nw-Nm2) /Nw = 1-Nm2/Nw. (This is done only because we are used to thinking about sex ratios as number of newborn boys divided by number of newborn girls, rather than the opposite.) Instead of (3), we can then write the relative difference between the Bs as

1. (Bw-Bm)/Bm ≈ (F1-F^(a)^1*)/ F^(a)^1* + (1-Nm2/Nw).

The ratio in the second term of (4), i.e. the number of men resident in the country at age 45 divided by the number of women in the two year younger cohorts resident in the country at age 45 (which is 1.018 when the male 1954-56 cohorts and female 1956-58 cohorts are considered), can be written as

1. Nm2/Nw = Nm2’/Nw2’ ∙ Nw2’/Nw’ ∙ (Nm2/Nm2’)/(Nw/Nw’).

The apostrophe refers to persons born in Norway. Nm2’/Nw2’ is the sex ratio among Norwegian-born individuals still resident in the country at age 45 and from the three oldest cohorts within the cohort group (which is 1.027 when the attention still is on the oldest cohort group). This ratio is the result of the sex-ratio at birth (1.047), differential survival (if the dead had been excluded, the ratio would have been 1.021) and emigration (a component which only changes the ratio from 1.021 to 1.027). The second term (Nw2’/Nw’) is the inverse of the increase in the number of women born in Norway and still resident in the country at age 45 from the oldest to the youngest three-year cohorts within the cohort group that is considered. It is 0.998 for the oldest cohort group (i.e., when the number of women born 1954-56 is compared with the number of women born 1956-58). Deaths and emigration do not contribute, because the ratio is 0.998 also if everyone is considered regardless of residence at age 45, and if the dead are excluded. The third term reflects the contribution of immigrants to the population of men (1.089) divided by the corresponding number for women (1.096), which is 0.994.

Going back to equation (4), F1 and F^(a)^1* are, of course, not equal in reality. However, the difference may often be quite small, not least because they overlap so strongly. For example, when the focus is still on the oldest cohort group, about 40% of both terms are births to women born in 1956-58 and men born in 1954-56, i.e. births that are included in both F1 and F^(a)^1*. In fact, F^(a)^1* is only 1.0% larger than F1, while it is 0.1% smaller in the younger cohort group (1971-73 and 1969-71).

To elaborate on this for the older cohort group, F1 includes all first births among women born in 1956-58 (let us call that cohort kw) where the father can be from any cohort, while F^(a)^1* includes all first births among men born in cohorts 1954-56 (let us call that cohort km) where the mother can be from any cohort – with the usual additional restrictions on age at birth and residence. F1 and F^(a)^1* are equal if, for example, for all values of x (which can be any positive or negative integer), the number of births to previously childless women and men born in kw+x and km, respectively, are the same as the number of births to previously childless women and men born in kw and km-x. This will be the case if there is a constant rise or decline over cohorts in the number of women who have a first child with childless men, and a stable pattern in the distribution of the age difference between the parents.

It turns out that the changes in the first and the last product term in (5) contribute little to the change in Bw-Bm from the older cohort group (1956-58 and 1954-56) to the younger (1971-73 and 1969-71). It is primarily the change in the middle term, which refers to the size of the male vs two year younger female cohorts among residents at age 45, that matters. The change in this relative cohort size is in turn largely a result of the fertility development, rather than deaths and emigration (see details in Notes to the main text).

Also the difference between, for example, Cw and Cm is affected by the sizes of the male and female birth cohorts. Indeed, (Cw-Cm)/Cm can be written just as (Bw-Bm)/Bm in equation (4), except that F1 and F^(a)^1* are substituted by F2 and H^(a)^1*, respectively. However, the difference between F2 and H^(a)^1* in the older cohort group (men born 1954-56 and women born 1956-58) is 6%, and thus contributes much more than the difference between F1 and F^(a)^1*, which is 1%. In other words, the contribution from the male relative to female cohort size constitutes a smaller proportion of Cw-Cm than of Bw-Bm in this particular case, and the same pattern may be seen in many other situations. Furthermore, because the Bs tend to be so large compared to the Cs and the other components (most people have their first child with a previously childless person), the absolute contribution from the relative cohort size to Bw-Bm (1.8%*Bm) is larger than its absolute contribution to Cw-Cm (1.8%*Cm) and the other components.

**Supplementary Table S1. Number of births in various categories**

Number of births to women born 1956-58, by characteristics of the father

|  |  | Number of births | Number of births where the father was 45 or younger at time of birth | | | | | Number of births where the father was older than 45 at time of birth | Number of births where the father was not identified |
| --- | --- | --- | --- | --- | --- | --- | --- | --- | --- |
|  |  |  | Father lived in Norway at age 45 | | | Father did not live in Norway at age 45 | |  |  |
|  |  |  | Father had no older children | | Father had older children | Father had no older children | Father had older children |  |  |
|  |  |  |  | Father born  1954-56 |  |  |  |  |  |
| Women born 1956-58 who lived in Norway at age 45  Nw=94212 | First births | F = 83433 | F1 = 70484 | F1* = 28709 | F2 = 6606 | F3 = 2522 | F4 = 310 | F5 = 868 | F6 = 2643 |
|  | Relative to Nw | Sw = 0.886 | Bw = 0.748 |  | Cw = 0.070 | Dw1 = 0.027 | Dw2 = 0.003 | Dw3 = 0.009 | Dw4=0.028 |
|  |  | | | | | | | | |
|  | Higher-order births | H = 110457 | H1 = 6737 | H1*= 1616 | H2 = 97300 | H3 = 309 | H4 = 2479 | H5 = 2503 | H6 = 1129 |
| Women born 1956-58 who did not live in Norway at age 45 | First births | Fx = 5661 | Fx1 = 1510 | Fx1* = 548 | Fx2 = 216 | Fx3 = 2643 | Fx4 = 83 | Fx5 = 82 | Fx6 = 1127 |
|  | Higher-order births | Hx = 5150 | Hx1 = 172 | Hx1* = 43 | Hx2 = 1549 | Hx3 = 93 | Hx4 = 2568 | Hx5 = 148 | Hx6 = 620 |

Number of births where the father was 45 or younger at time of birth, lived in Norway at age 45, and had no older children, by characteristics of the mother

|  |  | Women born 1956-58 | | | Women born in other cohorts | | Women born in any cohort | | |
| --- | --- | --- | --- | --- | --- | --- | --- | --- | --- |
|  |  |  |  | Father born 1954-56 |  | Father born 1954-56 |  | Father born 1954-56 | |
|  |  |  | Relative to Nm2 |  |  |  |  |  | Relative to Nm2 |
| Women who lived in Norway at age 45 | First births | F1 = 70484 | 0.735 | F1* = 28709 | F^(o)^1 = 1140636 | F^(o)^1* = 42485 | F^(a)^1 = 1211120 | F^(a)^1* = 71194 | Bm = 0.742 |
|  | Higher-order births | H1 = 6737 | 0.070 | H1* = 1616 | H^(o)^1= 197577 | H^(o)^1* = 4625 | H^(a)^1 = 114314 | H^(a)^1* = 6241 | Cm = 0.065 |
| Women who did not live in Norway at age 45 | First births | Fx1 = 510 | 0.016 | Fx1* = 548 | Fx^(o)^1 = 23962 | Fx^(o)^1* = 1022 | Fx^(a)^1 = 25472 | Fx^(a)^1* = 1570 | Dm1 = 0.016 |
|  | Higher-order births | Hx1 = 172 | 0.002 | Hx1* = 43 | Hx^(o)^1= 2094 | Hx^(o)^1* = 123 | Hx^(a)^1 = 2266 | Hx^(a)^1* = 166 | Dm2 = 0.002 |
|  |  |  |  |  |  |  |  |  |  |
| Sum |  | S1 = 78903 | 0.823 | S1* = 30916 | S^(o)^1 = 1274269 | S^(o)^1*= 48255 | S^(a)^1 = 1353172 | S^(a)^1* = 79171 | 0.826 |
|  |  |  |  |  |  |  |  |  |  |
| Unidentified mother |  |  |  |  |  |  |  | U = 250 | Dm4 = 0.003 |
| Sum |  |  |  |  |  |  |  | S 79421 | Sm = 0.828 |

Number of men born 1954-56 who lived in Norway at age 45: Nm2 = 95900

Notes: Twins or higher-order multiple births are counted as only one birth. “Lived in Norway at age 45” means “lived in Norway at the end of the year when they turned 45 or, if born after 1973, the end of 2018”.
